# Novel engineered B lymphocytes targeting islet-specific T cells inhibit the development of type 1 diabetes in non-obese diabetic Scid mice

**DOI:** 10.3389/fimmu.2023.1227133

**Published:** 2023-09-04

**Authors:** Dawei Chen, Dimitri Kakabadse, Sigal Fishman, Hadas Weinstein-Marom, Joanne Davies, Joanne Boldison, Terri C. Thayer, Li Wen, Gideon Gross, F. Susan Wong

**Affiliations:** ^1^ Diabetes Research Group, Division of Infection and Immunity, Systems Immunity University Research Institute, Cardiff University School of Medicine, Cardiff University, Cardiff, United Kingdom; ^2^ Laboratory of Immunology, MIGAL, Kiryat Shmona, Israel; ^3^ Department of Biotechnology, Tel-Hai College, Upper Galilee, Israel; ^4^ Section of Endocrinology, Internal Medicine, School of Medicine, Yale University, New Haven, CT, United States

**Keywords:** type 1 diabetes, NOD mice, regulatory B cells, CD4^+^ T cells, CD8^+^ T cells

## Abstract

**Introduction:**

In this study, we report a novel therapeutic approach using B lymphocytes to attract islet-specific T cells in the non-obese diabetic (NOD) mouse model and prevent the development of autoimmune diabetes. Rather than using the antibody receptor of B cells, this approach utilizes their properties as antigen-presenting cells to T cells.

**Methods:**

Purified splenic B cells were treated with lipopolysaccharide, which increases regulatory B (Breg) cell function, then electroporated with mRNA encoding either chimeric MHC-I or MHC-II molecules covalently linked to antigenic peptides. Immunoregulatory functions of these engineered B cells (e-B cells) were tested by *in vitro* assays and *in vivo* co-transfer experiments with beta-cell-antigen-specific CD8^+^ or CD4^+^ T cells in NOD.Scid mice, respectively.

**Results:**

The e-B cells expressing chimeric MHC-I-peptide inhibited antigen-specific CD8^+^ T-cell cytotoxicity *in vitro*. The e-B cells expressing chimeric MHC-II-peptide induced antigen-specific CD4^+^ T cells to express the regulatory markers, PD-1, ICOS, CTLA-4, Lag3, and Nrp1. Furthermore, e-B cells encoding the chimeric MHC-I and MHC-II peptide constructs protected NOD.Scid mice from autoimmune diabetes induced by transfer of antigen-specific CD8^+^ and CD4^+^ T cells.

**Discussion:**

MHC–peptide chimeric e-B cells interacted with pathogenic T cells, and protected the host from autoimmune diabetes, in a mouse model. Thus, we have successfully expressed MHC–peptide constructs in B cells that selectively targeted antigen-specific cells, raising the possibility that this strategy could be used to endow different protective cell types to specifically regulate/remove pathogenic cells.

## 1 Introduction

Type 1 diabetes (T1D) is a multifactorial autoimmune disorder in which autoreactive CD8^+^ and CD4^+^ T lymphocytes, together with B lymphocytes, infiltrate pancreatic islets of Langerhans, leading to the loss of insulin-producing β cells. Both CD4^+^ helper and CD8^+^ cytotoxic T cells are involved in the pathogenic process. CD8^+^ T cells are the major subset of lymphocytes that have a direct cytotoxic effect on the islets (1), while CD4^+^ T cells are often necessary for CD8 T cells to function, providing help, particularly the production of IL-2 (2).

However, there are other important immune cells involved in pathogenesis of T1D. B cells have multiple roles, including production of autoantibodies that serve as a biomarker to predict future development of diabetes (3). B lymphocyte-depletion studies using the anti-CD20 antibody, Rituximab, in people who have T1D have underlined the importance of B lymphocytes, beyond their production of autoantibodies. B lymphocyte-depleted individuals had a delayed decline of endogenous insulin production, measured by C-peptide (4, 5), and B cell depletion therapy in mouse models has both provided protection from diabetes when administered in prediabetic mice and reversed diabetes when administered after diabetes onset (6). Within the islets of people with T1D, B lymphocytes are present at the time of diagnosis of T1D (7) and have been identified within post-mortem samples of individuals who had recently developed T1D (8). There are increased numbers of both CD8^+^ T cells and B cells within pancreatic islets at a younger age of diagnosis (8), but their role in the affected tissue is not fully understood.

In recent years, a heterogeneous group of B cells have been identified to have regulatory function, and produce cytokines including IL-10, TGF-β, and IL-35 (9–12). They suppress immune responses, aid in generating other regulatory lymphocytes, and may also prevent the onset of diabetes in the non-obese diabetic (NOD) mouse (13, 14). Lipopolysaccharide (LPS)-stimulated B cells increase TGF-β production and these cells have prevented the adoptive transfer of diabetes in the NOD mouse (9–12). Furthermore, anergic CD40^+^ IL-10-producing B cells have been identified in the pancreas of mice that have not developed diabetes and these B cells may have a protective role (15).

Chimeric antigen receptor (CAR)-expressing T cells have been successfully used for the treatment of cancer (16) and strategies based on this development of CAR constructs to alter regulatory T cells have been tested in model systems (17–19). We have previously shown that a different strategy of expressing a modified CAR on CD8^+^ T cells induces the gene-modified CD8^+^ cytotoxic T cells to target antigen-specific CD8^+^ T cells, which can protect NOD mice from the development of autoimmune diabetes (20, 21). Recently, Yi and colleagues reported that CD8^+^ T cells expressing a myelin oligodendrocyte glycoprotein (MOG) peptide–MHCII CAR construct can efficiently deplete higher-affinity peptide-specific CD4^+^ T cells *in vivo* and prevent initiation of experimental autoimmune encephalomyelitis (EAE) (22). Thus, engineering immune cells expressing an MHC–peptide construct recognized by pathogenic T cells is attractive as an immunotherapy and has the potential to target pathogenic antigen-specific T cells, removing them specifically, without the likelihood of causing generalized immune suppression. Given the successful ability to express the constructs and selectively target antigen-specific cells, this raised the possibility that this strategy could be used to endow different protective cell types to regulate/remove pathogenic cells specifically.

Here, we present a novel proof of concept that B cells can be reprogrammed by electroporation with mRNA constructs encoding peptide/β_2_m (targeting CD8^+^ T cells) or peptide/MHC class II (targeting CD4^+^ T cells) to specifically attract and interact with autoreactive T cells of defined antigen specificity. Recognition of target peptide presented by the engineered B cells (e-B cells) reduced cytotoxicity of antigen-specific CD8^+^ T cells and induced regulatory markers on the antigen-specific CD4^+^ T cells. Furthermore, co-adoptive transfer of these e-B cells particularly regulated antigen-specific CD4^+^ T cells, reduced insulitis, and provided protection from diabetes in an adoptive transfer model of autoimmune diabetes.

## 2 Materials and methods

### 2.1 Mice

NOD mice, originally from the NOD/CaJ colony, BDC2.5 TCR transgenic mice (23), G9Cα^−/−^ TCR transgenic mice (24), and NOD.Scid mice were bred in-house at Cardiff University. All mice were housed in isolators or scantainers in a specific pathogen-free facility with a 12-h dark–light cycle and given food and water *ad libitum*. All experimental animal procedures were approved by the Cardiff University Biological Standards Committee, and carried out under UK Home Office License approval, in accordance with United Kingdom Animals (Scientific Procedures) Act, 1986 and associated guidelines.

### 2.2 B-cell isolation

Spleens from non-diabetic mice (6–10 weeks old) were homogenized and erythrocytes were lysed. B cells were isolated by negative selection using magnetic separation (Miltenyi Biotec, UK). The isolated B cells were stimulated for 24 h in the presence of 5 μg/ml of LPS (Sigma, UK) in complete Iscove’s Modified Dulbecco’s Medium (IMDM) (complete medium - IMDM with 5% fetal bovine serum + penicillin-streptomycin + L-glutamine + insulin-transferrin-selenium) at 37°C, 5% CO_2_ (25). Alternatively, B cells were stimulated with 5 μg/ml anti-CD40 monoclonal antibody (BioXCell, USA) and cultured in the same complete medium as above (25).

### 2.3 CD9^+^ B-cell enrichment

B cells were isolated and stimulated using LPS as described before. Activated B cells were harvested and stained with FITC-anti-CD9 antibody for 15-20 min. Labeled cells were washed with Dulbecco’s Phosphate Buffered Saline (DPBS), which contained 0.5% BSA and 2 mM EDTA and then incubated with anti-FITC beads (Miltenyi Biotec, UK) according to the manufacturer’s protocol. The bead-attached B cells were then separated by magnetic columns and checked for purity by flow cytometry.

### 2.4 mRNA *in vitro* transcription and delivery by electroporation

The genetic construct using MHC class I-insulin peptide chimeric construct (26–28), for insulin-reactive CD8^+^ T-cell interaction, incorporated modified insulin B15-23 peptide LYLVCGERV/hβ_2_m (20, 21). A non-relevant antigen construct, influenza nucleoprotein antigen (NP_50-57_, SDYEGRLI), was used as a control for antigen-specific recognition (26). For interaction with antigen-specific CD4^+^ T cells, a hybrid insulin-chromogranin A peptide (2.5HIP) sequence DLQTLALWSRMD (29) was utilized and a 2.5HIP peptide/I-A^g7^ construct was made, based on a previous construct incorporating BDC2.5 mimotope peptide/I-A^g7^ (30), with the 2.5HIP peptide substituted for the mimotope. The *pGEMT* vector for direct cloning of PCR products was from Promega (Madison, WI). Template DNA for *in vitro* transcription of mRNA was cloned into the pGEM4Z/GFP/A64 vector (Fishman et al, 2017) and was prepared as previously described (21). Construct mRNA was transcribed *in vitro* using the T7 mScript Standard mRNA Production System (Cell-Script, USA) to generate 5′-capped mRNA. Cells were washed twice with Opti-MEM medium (Gibco) and re-suspended in 200 μl of Opti-MEM containing the required amount of *in vitro*-transcribed mRNA (10–20 µg). Electroporation was performed with BTX Harvard Apparatus ECM830 (1 ms, 300 V) into B cells.

### 2.5 Flow cytometry and antibodies

Staining for extracellular or intracellular markers was performed as described previously (25). Briefly, for extracellular staining, the cells were incubated in TruStain (BioLegend, UK) for 10 min at 4°C to block the Fc receptors. Multi-parameter flow cytometry was carried out using the following antibodies: CD4-AF700 (RM4-5), CD44-BV650 (IM7), CD69-BV711 (H1.2F3), B220-BV785 (RA3-6B2), CD9-FITC (MZ3), CD62L-PE-Cy7 (MEL-14), LAP-PerCP-Cy5.5 (TW7-16B4), PD1-BV605 (29F.1A12), Lag3-PE-Dazzle594 (C9B7W), Nrp1-PerCP-Cy5.5 (3E12), CD86-PE (PO3), CD21-PE (7E9), and IgM-APC-Cy7 (H57-597) (all from BioLegend), as well as CD80-BV650 (16-10A1) and CD23-BV711 (B3B4) (both from BD Biosciences). Dead cells were excluded from the analysis using Live/Dead Viability Dye (Thermo Fisher).

For intracellular cytokine analysis, cells were treated with phorbol 12-myristrate-13-acetate (PMA) (50 ng/ml), ionomycin (500 ng/ml), and monensin (3 μg/ml) (all from Sigma-Aldrich) for 3 h. Fc receptors were blocked using TruStain and, after the extracellular staining, the cells were fixed using fixation/permeabilization according to the manufacturer’s instructions. The cells were then incubated with antibodies against intracellular markers, including IL10-APC (JES5-16E3, BD Biosciences) and IFNγ-PE-Cy7 (XMG1.2, BioLegend), or with appropriate isotype controls. Flow cytometry was performed on LSRFortessa (FACS Diva software) and analysis was performed using FlowJo software 10.8.1 (Treestar).

### 2.6 CD4^+^ T-cell proliferation assay

To analyze CD4^+^ T-cell proliferation *in vitro*, naïve BDC2.5 CD4^+^ T cells were isolated from BDC2.5 transgenic mouse splenocytes and labeled using CellTrace Violet (Life Technologies) according to the manufacturer’s instructions. The labeled CD4^+^ T cells were seeded with B cells, with or without construct, at a 1:1 ratio per well or 1:5 ratio per well ratio in 96-well U-bottomed plates, then cultured in RPMI complete medium (complete medium components as for B cells, with RPMI substituted for IMDM) at 37°C, 5% CO_2_ for 24 h. The proliferation of CD4^+^ T cells was assessed by the dilution of CellTrace Violet.

### 2.7 CD8^+^ T-cell cytotoxic assay

Cytotoxicity assays were performed using naïve G9Cα^−/−^ CD8^+^ T cells (10^5^) (hereafter referred to as G9 CD8 T cells), isolated from G9Cα^−/−^ TCR transgenic mouse splenocytes, co-cultured with PKH-labeled P815 target cells (10^4^) that were coated with INS_B15-23_ peptide (1 μg/ml and 5μg/ml) at an Effector : Target (E:T) ratio of 10:1 in 96-well U-bottomed plates. CD9^+^ or CD9^–^ e-B cells (10^5^), either mock-transfected or expressing INS_B15-23_/hβ_2_m construct, were added to the cytotoxicity assay using an equal number of e-B cells and CD8^+^ T cells. The cells were co-cultured at 37°C, 5% CO_2_, for 16 h. After incubation, cytotoxicity of CD8^+^ T cells towards the target cells was determined by the percentage of PKH+ dead cells, using flow cytometry.

### 2.8 Cytokine assays

Supernatants from cell culture assays were collected after 3 days. The secreted cytokines in the supernatant were measured by the Meso Scale Discovery (MSD) system according to the manufacturer’s (Meso Scale Diagnostics, LLC.) instructions and detected using MSD Sector Imager 6000.

### 2.9 *In vivo* adoptive transfer

NOD B cells were isolated, activated, and then transfected with mRNA. As a control, B cells were subjected to the electroporation procedure but with no added mRNA (Mock). For some of the transfers, the total B cells were separated to CD9^+^ and CD9^−^ B cells prior to transfection. To assess *in vivo* expression of the constructs, the e-B cells were adoptively co-transferred into 5- to 6-week-old NOD.Scid mice by intravenous injection into the tail vein. The mice were studied at 24 and 48 h for *in vivo* expression of the genetic constructs in the B cells.

The e-B cells transfected with the appropriate construct were also used for co-transfer with antigen-specific CD8^+^ or CD4^+^ T cells and tested for induction of diabetes. For the CD8^+^ T-cell co-adoptive transfer experiments to study diabetes induction/protection, G9Cα^−/−^ CD8^+^ T cells were isolated from the spleens of G9Cα^−/−^ mice (6–10 weeks old) and activated using plate-bound anti-CD3 (clone: 2C11, Bio X Cell) and anti-CD28 (clone37.51, Bio X Cell) antibodies for 48 h. The G9Cα^−/−^ CD8^+^ T cells were adoptively co-transferred with CD9^+^ or CD9^−^ e-B cells, expressing the INS_B15-23_/hβ2m construct, or mock-transfected, into 5- to 6-week-old NOD.Scid mouse recipients, by intravenous injection into the tail vein at an e-B cell-to-CD8^+^ T cell ratio of 1:1 (5 million/5 million cells) per mouse. Further experiments were performed using unseparated total e-B cells, co-transferred with G9Cα^−/−^ CD8^+^ T cells using a total B cell-to-CD8^+^ T cell ratio of 5:1 (25 million/5 million cells) per mouse.

For the CD4^+^ co-adoptive transfer experiments, BDC2.5 CD4^+^ T cells were isolated from the spleens of BDC2.5 mice (6–10 weeks old) and activated using bone-marrow-derived DCs (BMDCs) as APCs to present 2.5HIP peptide for 3 days. BMDCs were pre-stimulated for 16–18 h using LPS and washed in fresh medium three times before co-culture with BDC2.5 CD4^+^ T cells and 2.5HIP peptide. The total B cells or CD9^+^ or CD9^−^ B cells together with BDC2.5 CD4^+^ T cells were adoptively co-transferred at a B cell-to-CD4^+^ T cell ratio of 1:1 (5 million/5 million cells) per mouse.

The adoptively transferred recipient mice were monitored daily for glycosuria (Bayer Diastix). Following two consecutive positive readings, diabetes was confirmed by blood glucose levels >13.9 mmol/L.

### 2.10 Histology

The pancreas was fixed in paraformaldehyde lysine periodate buffer overnight and then infused with 10% sucrose in PB buffer, followed by 20% sucrose, as previously described (20, 21). The pancreas was then embedded in OCT and snap frozen for immunohistochemistry. Frozen sections, 10 μm thick, were stained with rat-anti-mouse CD4^+^, CD8^+^, B220, and anti-insulin antibodies. They were then detected with a Vector Red AP substrate kit (Vector Laboratories, Peterborough, UK) and streptavidin-alkaline phosphatase, followed by counterstaining with hematoxylin. Insulitis was assessed from two to three mice per group and 73–119 islets were blind scored. Scoring for insulitis is shown in the figure legend.

### 2.11 Statistical analysis

Unpaired Student’s *t*-tests were performed to compare data between groups. *χ* ^2^ test was performed for the analysis of insulitis. Log rank analysis was carried out for the adoptive transfer experiments. For all tests, *p* < 0.05 was considered statistically significant.

## 3 Results

### 3.1 The expression of an INSB_B15-23_/hβ_2_m construct in transfected B cells

**Figure 1:**
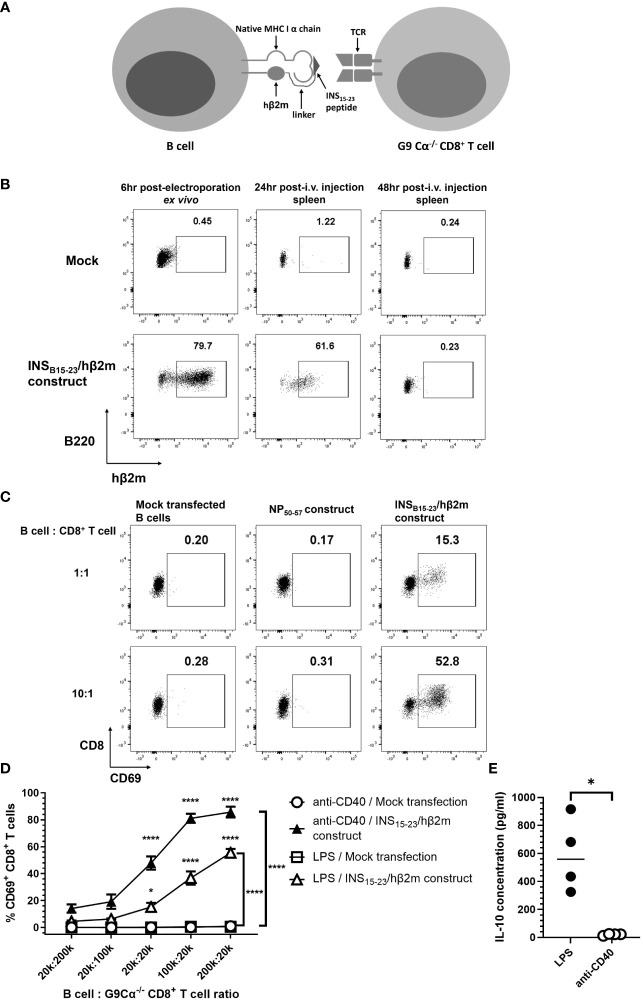
The expression of INS_B15-23_/hβ_2_m construct. **(A)** Scheme of the cell-surface expression of INS_B15-23_/hβ_2_m construct on e-B cells and the interaction with G9Cα^−/−^ CD8^+^ T cells. **(B)** The expression of INS_B15-23_ construct was tracked by extracellular staining of hβ_2_m. B cells from NOD mice were activated with 5 μg/ml LPS for 16–18 h Activated B cells were washed and electroporated without (Mock) or with mRNA of the INS_B15-23_/hβ_2_m construct, and rested for a minimum of 3 h at room temperature. After resting, 5×10^6^ cells/mouse of B cells were washed in saline and injected intravenously into NOD.Scid female mice. Spleen tissues of the recipient mice were collected 24 and 48 h post-injection. The plots are representative of three independent experiments, with six mice in total. **(C)** The expression of CD69, as an activation marker, on G9Cα^−/−^ CD8^+^ T cells co-cultured with NOD B cells with no construct (Mock) or non-relevant-antigen construct–influenza Nucleoprotein antigen (NP_50-57_) or expressing the INS_B15-23_/hβ2m construct. The B cells were electroporated without (Mock) or with the non-relevant NP_50-57_ construct or the INS_B15-23_/hβ2m construct and co-cultured with G9Cα^−/−^ CD8^+^ T cells at different ratios for 24 h. The numbers in these plots show the frequency of CD69^+^G9CD8^+^ T cells. The plots are representative of three independent experiments. **(D)** Line graphs showing the percentage of CD69^+^CD8^+^ T cells after 24 h of co-incubation with B cells expressing the INS_B15-23_/hβ_2_m construct or mock-transfected, which were pre-activated using LPS or anti-CD40 antibody. The two mock-transfected conditions were very close to each other and appear superimposed as there was no/little activation for either of these. The plots show mean ± SEM of three independent experiments, with seven mice in total. **(E)** Summary graph showing significant difference in IL-10 secretion between LPS-stimulated B cells and anti-CD40 stimulated B cells. B cells were stimulated and electroporated without construct. After 24 h culture, supernatants were collected and IL-10 concentration in the supernatant was detected by ELISA. Statistical analysis was performed using Mann–Whitney test. *p < 0.05, ****p < 0.0001.

To study the effects of pathogenic CD8^+^ T cells with well-characterized antigen specificity, we have employed insulin-specific pathogenic G9 CD8^+^ T cells recognizing the insulin B chain 15–23 peptide (31). These cells are among the earliest CD8^+^ T cells found in the pancreatic islets of the NOD mouse and G9Cα^−/−^ TCR transgenic T cells adoptively transfer diabetes to immunodeficient NOD.Scid mice very reliably (24). We expressed the previously described mRNA INS_B15-23_/hβ_2_m construct (20, 21) in NOD mouse splenocyte B cells (scheme in **Figure 1A**). This construct comprises a variant of the insulin B15–23 peptide, which binds H-2K^d^, substituted at position 9 (G9V) (20, 21), to generate a fusion protein of the insulin peptide, human β_2_-microglobulin (hβ_2_m) and the CD3-ζ chain. Thus, the B cells expressing the INS_B15-23_/hβ_2_m chimeric polypeptide construct will also express the endogenous H-2K^d^ heavy chain that will pair with the construct. This molecule is designed to present antigen to insulin B15–23-reactive T cells. To demonstrate the expression of this engineered INS_B15-23_/hβ_2_m construct, we stimulated the B cells using LPS (well known to activate B cells) for 16–18 h prior to electroporation, followed by labeling the cells with an anti-hβ_2_m antibody enabling detection of the expression of the INS_B15-23_/hβ_2_m construct by flow cytometry. The construct was expressed by approximately 80% of the B cells (designated e-B cells) 6 h after electroporation (**Figure 1B**). A large proportion of the e-B cells were also found in the splenocytes of NOD.Scid recipient mice 24 h after adoptive transfer of the e-B cells (**Figure 1B**). However, the construct expression in the transferred B cells was not detectable in the spleen by 48 h.

### 3.2 The functional expression of the INS_B15-23_/hβ2m construct in e-B cells activates insulin-reactive CD8^+^ T cells

To investigate the biological function of the INS_B15-23_/hβ_2_m construct in e-B cells, we co-cultured the e-B cells and insulin-reactive G9 CD8^+^ T cells from G9Cα^−/−^ TCR transgenic NOD mice, which recognize the INS_B15-23_ peptide presented by H-2-K^d^ (24). These transgenic G9 CD8^+^ T cells, which recognized the INS_B15-23_/hβ_2_m construct on the e-B cells, were activated, as shown by the dose-dependent upregulation of the early activation marker CD69 (**Figure 1C**). This T-cell activation was not seen when the e-B cells were mock-transfected or transfected with a non-relevant antigen construct control (**Figure 1C**). Thus, our results indicated that the INS_B15-23_/hβ_2_m construct induced on NOD B cells was functional and these e-B cells expressing the INS_B15-23_/hβ_2_m construct interacted with and activated the INS_B15-23_ antigen-specific G9 CD8^+^ T cells.

To further investigate the function of the e-B cell in activating antigen-specific CD8^+^ T cells, we pre-activated e-B cells with the innate immune stimulator, LPS, and compared this with e-B cell activation using the adaptive immune stimulator, anti-CD40, prior to the co-culture with G9 CD8^+^ T cells. We demonstrated that INS_B15-23_/hβ_2_m construct-expressing e-B cells, stimulated by either LPS or anti-CD40, strongly activated INS_B15-23_-specific G9 CD8^+^ T cells, with a greater response to the anti-CD40-activated e-B cells (**Figure 1D**). However, LPS stimulation induced a high level of IL-10 production, which was not seen following anti-CD40 stimulation (**Figure 1E**). As our aim was to enrich the B cells for suppressive function, we chose to use LPS to stimulate the e-B cells to express the INS_B15-23_/hβ_2_m construct and test the suppressive function, for all future experiments.

### 3.3 Expression of 2.5HIP/I-A^g7^ construct in e-B cells activates 2.5HIP-specific diabetogenic CD4^+^ T cells

**Figure 2:**
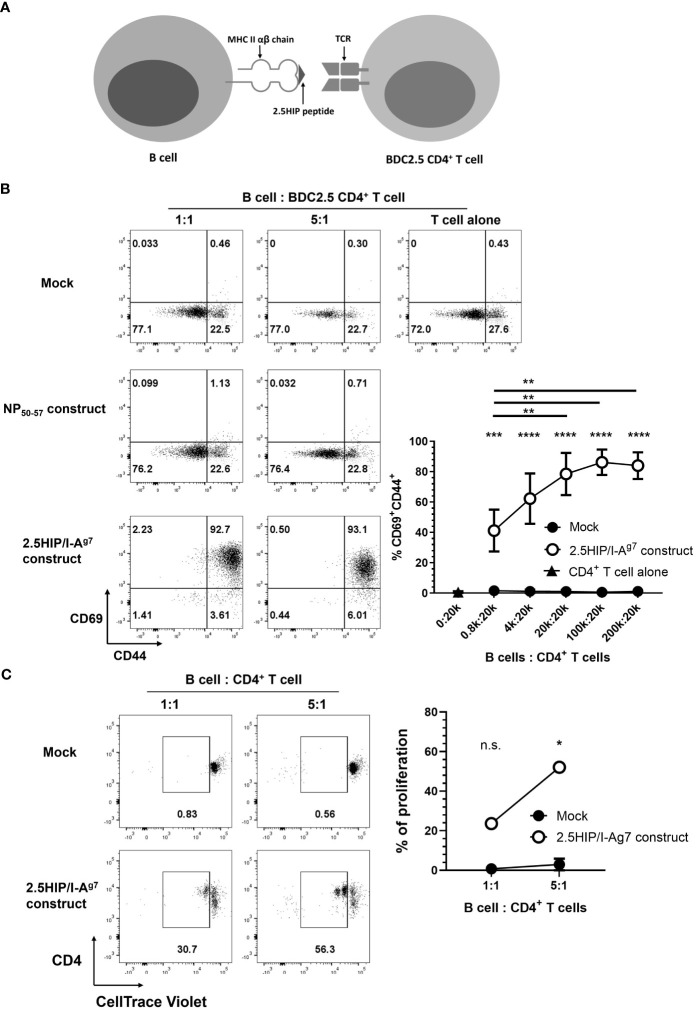
Engineered B cells (e-B cells) expressing 2.5HIP/I-A^g7^ construct activate BDC2.5 CD4^+^ T cells. **(A)** The scheme of the 2.5HIP/I-A^g7^ construct expressed on the cell surface of e-B cells and the interaction with BDC2.5 CD4^+^ T cells. **(B)** The expression of CD69 and CD44, as activation markers, on BDC2.5 CD4^+^ T cells after 24 h co-culture with e-B cells transfected without (mock) or with non-relevant or 2.5HIP/I-A^g7^ construct. The B cells were electroporated without (mock) or with the 2.5HIP/I-A^g7^ construct, or a non-relevant antigen (NP50-57) construct. e-B cells were co-cultured with BDC2.5 CD4^+^ T cells at different ratios for 24 h. The plots are representative of three independent experiments and the line graph is the mean ± SEM of the three experiments. Statistical analysis was performed using two-way ANOVA and multiple comparisons. Significance shown indicates the result between mock-transfected and 2.5HIP/I-A^g7^ construct group. ***p < 0.001, ****p < 0.0001. **(C)** Proliferation of BDC2.5 CD4^+^ T cells co-cultured with e-B cells transfected without (control) or with 2.5HIP/I-A^g7^ construct for 24 h. BDC2.5 CD4^+^ T cells were labeled with CellTrace Violet and co-cultured with e-B cells at B cell:T cell 1:1 and 5:1 ratios. The frequency of gated proliferated cells (in duplicate), with loss of CellTrace violet indicating proliferation, is plotted in the line-graph, shown as the mean ± SEM of three independent experiments. Statistical analysis was done by Student’s *t* test. *p < 0.05. **p <0.01.

To allow us to study pathogenic CD4^+^ T cells, we have employed BDC2.5 CD4^+^ T cells, which were the first characterized highly pathogenic cloned CD4^+^ T cells (32), recognizing a novel insulin-chromogranin A hybrid peptide (2.5HIP) (29). Cells from the BDC2.5 TCR transgenic mouse also aggressively adoptively transfer diabetes to NOD.Scid mice. To test for effects on 2.5HIP-specific CD4^+^ T cells, we generated a construct with a hybrid insulin-chromogranin A peptide (2.5HIP) (29), conjugated to the MHC class II α and β chain of I-A^g7^, the specific antigen recognized by pathogenic BDC2.5 CD4^+^ T cells (2.5HIP/I-A^g7^) (**Figure 2A**), modified from a previously developed construct (30). We transfected this 2.5HIP/I-A^g7^ construct into LPS-stimulated B cells and co-cultured these e-B cells with 2.5HIP/I-A^g7^-specific BDC2.5 CD4^+^ T cells. e-B cells, either mock-transfected or transfected with a non-relevant construct, were used as controls. After 24 h, over 90% of the BDC2.5 CD4^+^ T cells co-cultured with the 2.5HIP/I-A^g7^ construct-expressing e-B cells upregulated the activation markers CD69 and CD44, compared with fewer than 1% control e-B cells (**Figure 2B**). Moreover, the BDC2.5 CD4^+^ T cells proliferated, as indicated by dilution of the CellTrace Violet dye, on recognition of the 2.5HIP/I-A^g7^ construct expressed on the e-B cells, at two different ratios of 2.5HIP/I-A^g^ e-B cells to BDC2.5 CD4^+^ T cells, increasing at the higher ratio, whereas no proliferation was seen at either ratio of mock-transfected B cells (**Figure 2C**). These data indicated that the 2.5HIP/I-A^g7^ antigen-specific CD4^+^ T cells specifically recognized and were activated by the 2.5HIP/I-A^g7^ construct on the e-B cells. Although the expression of the construct was relatively short lived, as it is an mRNA construct, the MHC–peptide complex was expressed for a sufficient length of time to stimulate the BDC2.5 CD4^+^ T cells to continue proliferation, which is seen for at least 3 days of culture (**Supplementary Figure 1**), with concomitant changes also in the surface markers (see below).

### 3.4 IL-10^+^ producing e-B cells are enriched using CD9-positive selection and suppress CD8^+^ T-cell cytotoxicity

**Figure 3:**
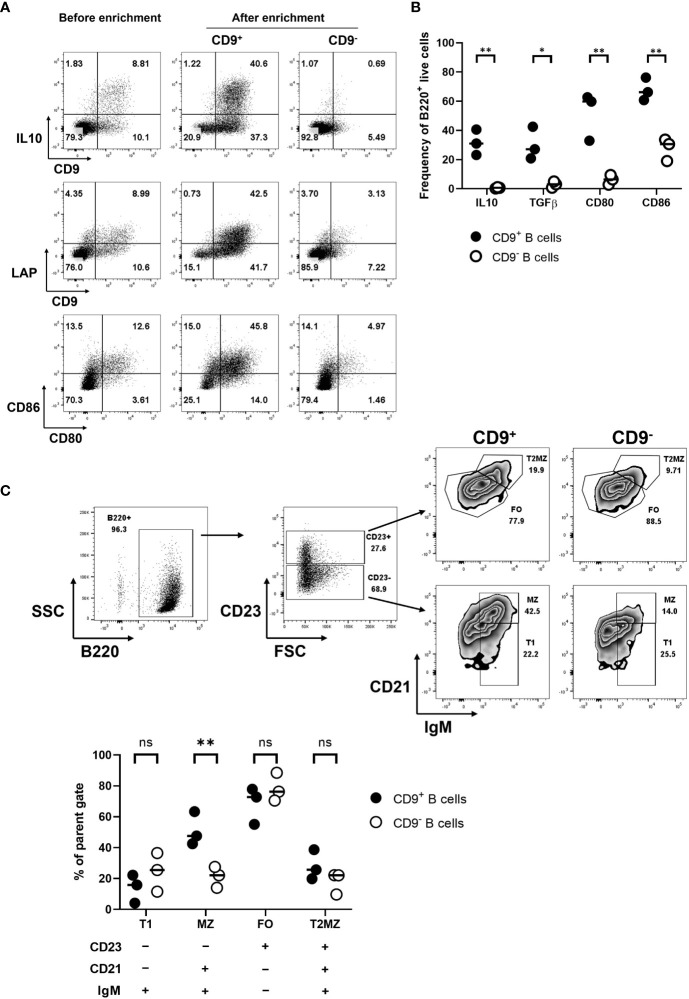
Enriched IL-10-competent CD9^+^ B cells. **(A)** CD9^+^ B cell enrichment. B cells were isolated, then stimulated with LPS for 18 h. Activated B cells were harvested and stained using FITC-conjugated anti-CD9 antibodies. CD9^+^ B cells were positively selected using anti-FITC magnetic beads. CD9^+^ and CD9^−^ populations were then restimulated using PMA, ionomycin, and monensin for 3.5 h to perform intracellular staining for cytokines, followed by staining with the appropriate antibodies for IL-10 and extracellular LAP. **(B)** A scatter plot shows the differential expression of intracellular IL-10 and TGF-β for the CD9^+^ and CD9^−^ B cell populations; the individual points on the graph show results from three independent experiments. **(C)** Characterization of CD9^+^ and CD9^−^ B cell populations, showing distribution within MZ, T1, T2MZ, and FO zones (36), from three independent experiments. Statistical analysis was performed by multiple comparisons. *p <0.05, **p < 0.01. ns not statistically significant.

Aiming to develop regulatory B-cell (Breg) immunotherapy to disarm and/or disable antigen-specific pathogenic T cells, we tested a strategy to further enhance the diabetes-protective potential of the e-B cells. Several B-cell subsets have regulatory potential (33, 34), including B cells expressing high levels of regulatory cytokines, IL-10 and/or TGF-β. While there is no unique identifying feature of regulatory B cells, CD9 has been shown to be a marker of murine IL-10-competent B cells (35). Using LPS, we stimulated total B cells, or CD9^+^ B cells enriched by positive selection, and assessed IL-10 expression. Our results showed that the CD9^+^ B cells were enriched for IL-10^+^ cells (**Figure 3A**), and a higher level of latency-associated peptide (LAP) that releases active TGF-β (**Figure 3B**), as well as higher levels of CD80 and CD86, compared with the CD9^−^ population (**Figures 3A, B**). We further characterized the CD9^+^ B cells to be CD23^-^CD21^+^IgM^+^ marginal-zone-like B cells (**Figure 3C**), which have previously been shown to contain Breg (35). Thus, by enriching for a CD9^+^ B cell subset, we were enriching for Breg.

**Figure 4:**
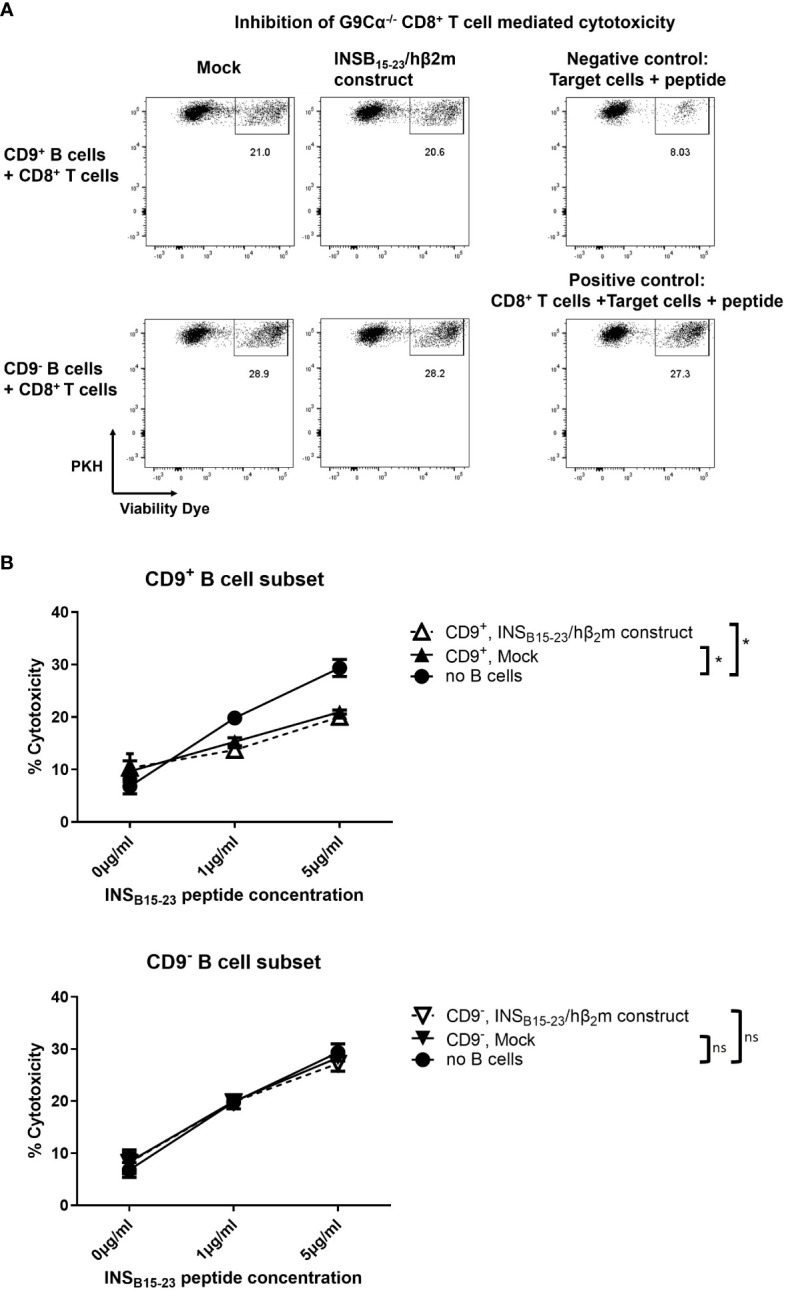
CD9^+^ B cells inhibit the cytotoxicity of CD8^+^ T cells *in vitro*. **(A)** Representative flow cytometry plots demonstrating cytotoxicity assay using naïve G9Cα^−/−^ CD8^+^ T cells co-cultured with PKH-labeled P815 target cells coated with INS_B15-23_ peptide (1 or 5 μg/ml) at an effector:target ratio of 10:1. CD9^+^ or CD9^–^ e-B cells (mock-transfected or expressing INS_B15-23_/hβ_2_m construct) were added to the cytotoxicity assay using an equal number of e-B cells and CD8^+^ T cells. The controls did not have added e-B cells; the negative control was target cells alone and the positive control was G9Cα^−/−^ CD8^+^ T cells together with target cells. The cells were co-cultured at 37°C, 5% CO_2_, for 16 h. After incubation, cells were collected and cytotoxicity was determined by the percentage of dead PKH-labeled P815 target cells. **(B)** Summary of cytotoxicity assays with added CD9^+^ or CD9^−^ e-B cell subsets to demonstrate effect on cytotoxicity by G9Cα^−/−^ CD8 T cells towards INS_B15-23_ peptide-coated target cells. The data are a summary of three independent experiments. Statistical analysis was performed by multiple comparisons. *p < 0.05. ns, not statistically significant.

Next, we tested if CD9^+^ e-B cells regulated CD8^+^ T cells. We enriched for CD9^+^ B cells and tested their regulatory activity in a CD8^+^ T-cell cytotoxicity assay *in vitro*. The CD9^+^ B-cell subset suppressed the cytotoxicity of G9Cα^−/−^ CD8^+^ T cells towards the INS_B15-23_ peptide-presenting target cells, whereas the CD9^−^ B-cell subset did not (**Figure 4A**). We tested CD9^+^ and CD9^−^ e-B cells expressing the INS_B15-23_/hβ_2_m construct, against targets coated with 1 and 5 μg INS_B15-23_ peptide concentrations in the assay. At both INS_B15-23_ peptide concentrations, CD9^+^ but not CD9^−^ e-B cells suppressed cytotoxicity of the G9Cα^−/−^ CD8^+^ T cells, but there was no additional enhancement of the suppressive potential when the antigen-specific construct was expressed in the e-B cells (**Figure 4B**). In this experiment, the *in vitro* setup conditions brought the CD9^+^ B cells into close proximity to the G9Cα^−/−^ CD8^+^ T cells, irrespective of the presence of the construct, which would allow for any intrinsic suppressive effect of CD9^+^ B cells to be observed. In summary, both CD9^+^ and CD9^–^ e-B cells can express the INS_B15-23_/hβ_2_m construct, but only CD9^+^ B cells had a direct suppressive effect on CD8^+^ T-cell activity, under the culture conditions tested.

### 3.5 IL-10-producing CD9^+^ e-B cells induce 2.5HIP/I-A^g7^-specific BDC2.5 CD4^+^ T cells to upregulate the regulatory Lag3 and Nrp1 markers

Next, we investigated the effect of antigen-specific e-B cells on CD4^+^ T cells. Using the CD4^+^ T-cell-specific 2.5HIP/I-A^g7^ construct (as shown in **Figure 2**), we investigated the capacity of the BDC2.5HIP/I-A^g7^ construct-expressing e-B cells to induce regulatory markers on BDC2.5 CD4^+^ T cells. We cultured naïve BDC2.5 CD4^+^ T cells with 2.5HIP/I-A^g7^ e-B cells (CD9^+^, CD9^−^, and total B cells) and assessed the expression of activation and immune regulatory markers, including CD62L, PD-1, Lag3, Nrp1, CTLA-4, ICOS, and FoxP3 on BDC2.5 CD4^+^ T cells during the 3-day-culture.

**Figure 5:**
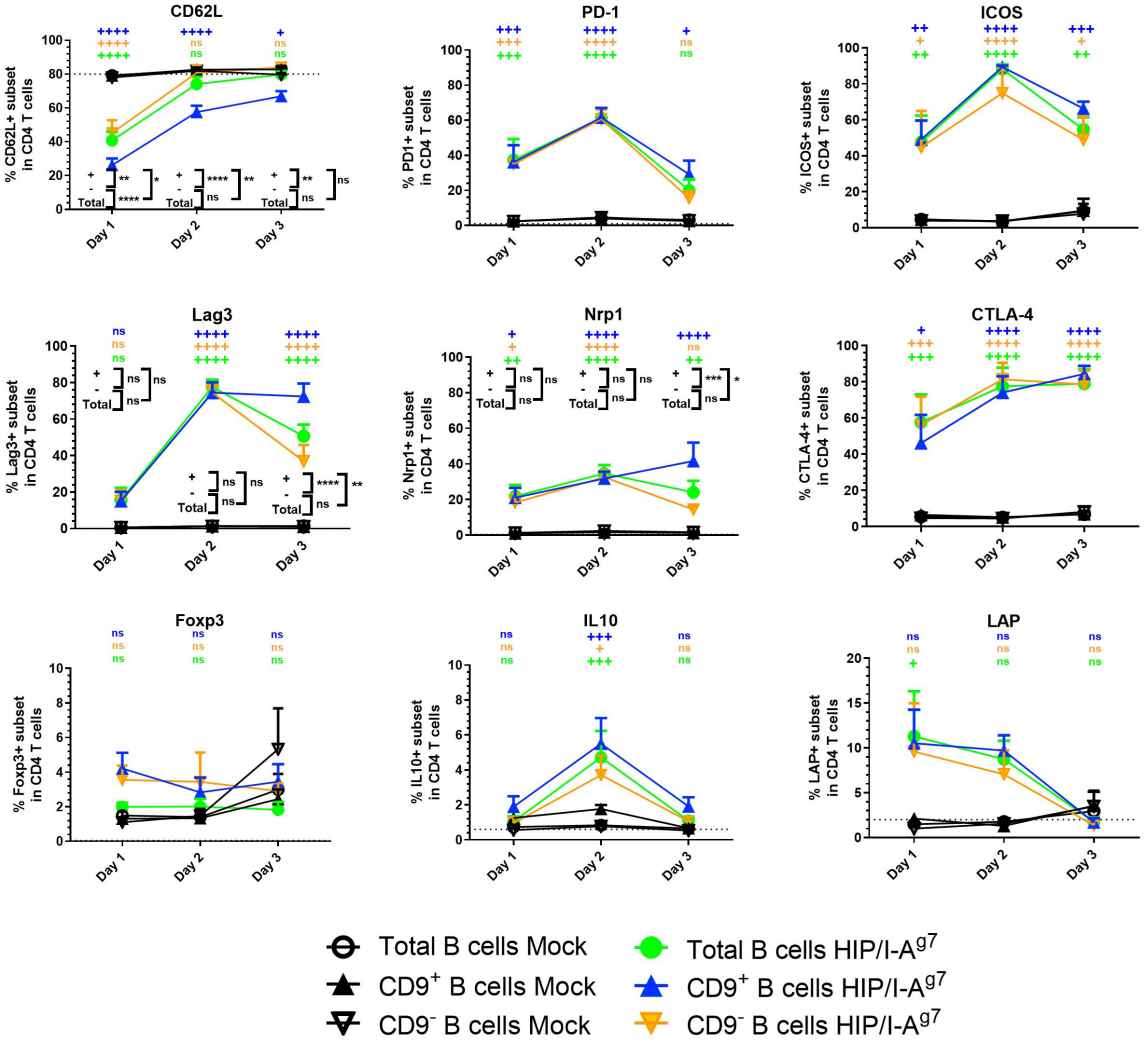
Expression of cell surface markers and intracellular cytokines in BDC2.5 CD4^+^ T cells co-cultured with e-B cells transfected with or without 2.5HIP/I-A^g7^ construct. The percentage of cell surface markers (CD62L, PD-1, Lag3, Nrp1, CTLA-4, ICOS, FoxP3, and LAP) and intracellular cytokine (IL-10) in the BDC2.5 CD4^+^ T cells co-cultured with CD9^+^, CD9^−^, or total e-B cells transfected with 2.5HIP/I-A^g7^ construct or without (mock-transfected). Naïve BDC2.5 CD4^+^ T cells were co-cultured with e-B cells with or without the 2.5HIP/I-A^g7^ construct at a 1:1 ratio for 3 days. Cells were treated using PMA, ionomycin, and monensin for 3.5 h before harvest for intracellular staining of cytokines. The graphs show the percentage expression of each of the indicated markers in the CD4^+^ T cells. These data show mean values ± SEM of five independent experiments. Statistical analysis was performed using two-way ANOVA, with multiple comparisons for the difference between time points, and multiple *t*-tests were used to compare the difference between e-B cell types. ns non-significant, +*p* < 0.05, ++*p* < 0.01, +++*p* < 0.001, ++++*p* < 0.0001, comparisons between 2.5HIP/I-A^g7^ construct vs. mock transfection within each B-cell group and are shown in black on the figure. Where there was no statistical significance (ns) between the 2.5HIP/I-A^g7^ construct vs. mock transfection within each B-cell group, at each time point, as for PD-1, ICOS, CTLA-4, Foxp3, IL-10, and LAP, these were performed but are not shown on the graph. *p < 0.05, **p < 0.01, ***p < 0.001, ****p < 0.0001, comparisons between different B-cell groups with 2.5HIP construct and are shown in color on the figure. All comparisons that showed any statistical significance are shown on the graph. ns, not statistically significant.

Over 72 h, CD62L expression remained high in the BDC2.5 CD4^+^ T cells that were co-cultured with mock-electroporated B cells (no construct), suggesting that the BDC2.5 CD4^+^ T cells were not activated, whereas the frequency of CD62L on CD4^+^ T cells cocultured with each of the 2.5HIP/I-A^g7^ e-B cells was decreased on Day 1, indicating the activation of CD4^+^ T cells (**Figure 5**). However, the level of CD62L in the CD9^+^ B cell co-culture group remained significantly lower than the other groups on Day 2, suggesting a different pattern of BDC2.5 CD4^+^ T-cell activation on interaction with the CD9^+^ e-B cells.

Importantly, the immune regulatory markers tested on the BDC2.5 CD4^+^ T cells were upregulated and some peaked by Day 2 of culture (**Figure 5**), although in the cultures with CD9^+^ e-B cells, the levels of Lag3 and Nrp1 were maintained, whereas in the cultures with engineered CD9^−^ and total e-B cells, the levels waned by Day 3 (**Figure 3**). Expression of CTLA-4 and ICOS increased compared with expression on the BDC2.5 CD4^+^ T cells when mock-transfected T cells were used, but these did not differ between the three subsets of e-B cells. FoxP3 also showed a small increase, but the expression differences were not statistically significant.

We further analyzed the BDC2.5 CD4^+^ T cells for IL-10 and LAP by intracellular staining after culturing with the different groups of e-B cells. The BDC2.5 CD4^+^ T cells cultured with 2.5HIP/I-A^g7^ e-B cells, regardless of CD9 expression, appeared to express higher IL-10 (on Day 2) and LAP (Days 1 and 2) than mock-transfected B cells (**Figure 5**).

**Figure 6:**
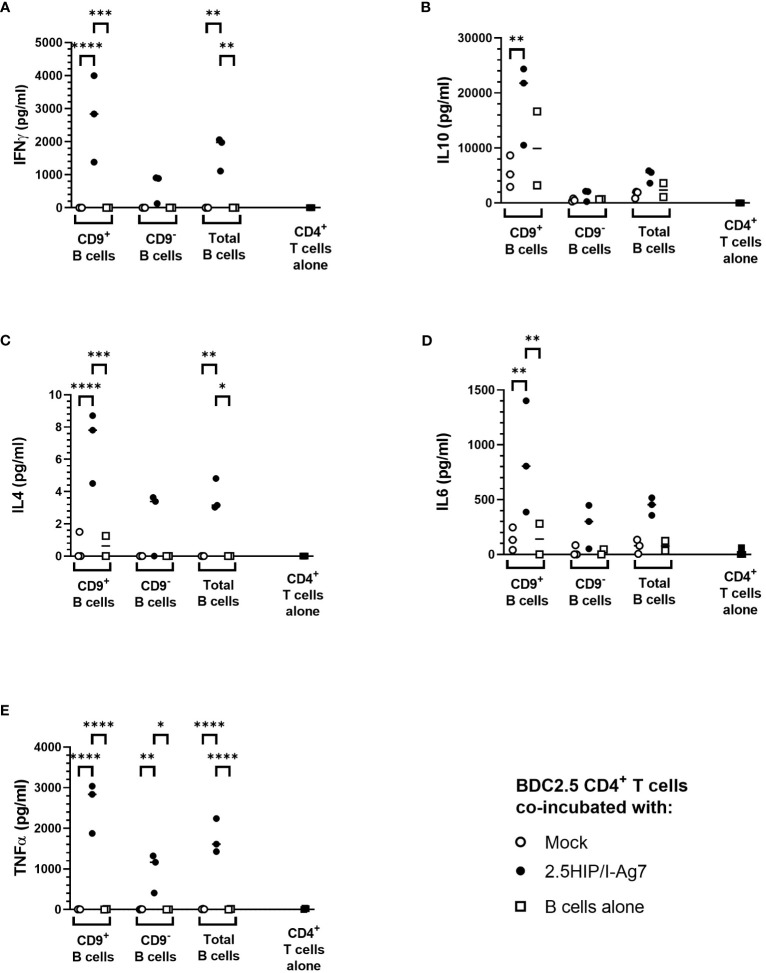
Cytokine production after co-culture of e-B cells and antigen-specific BDC2.5 CD4^+^ T cells. Cytokine levels in supernatant from e-B cells that have been either Mock or 2.5HIP/I-A^g7^ transfected and cultured with BDC2.5 CD4^+^ T cells. Naïve BDC2.5 CD4^+^ T cells were co-cultured with e-B cells with or without the 2.5HIP/I-A^g7^ construct at a 1:1 ratio for 3 days. Supernatants were collected on Day 3 and analyzed using MSD. The dotted line shows levels of cytokines from CD4 T cells alone. **(A)** IFN-γ. **(B)** IL-10. **(C)** IL-4. **(D)** IL-6. **(E)** TNF-α. Statistical analysis was performed using two-way ANOVA with multiple comparisons. Significance was determined by the *p* values using multiple comparisons between Mock and HIP/I-A^g7^ groups with the same cell types. *p < 0.05, **p < 0.01, ***p < 0.001, ****p < 0.0001.

However, when we assessed secreted pro-inflammatory (IFN-γ, IL-6, and TNF-α) and anti-inflammatory (IL-10 and IL-4) cytokines in the cultured supernatants, we found that the overall levels of these cytokines in the CD9^+^ 2.5HIP/I-A^g7^ construct-expressing e-B cell group were higher than the CD9^−^ 2.5HIP/I-A^g7^ construct-expressing e-B cell group (**Figure 6**). These results are also expressed as a ratio of inflammatory cytokines to IL-10, with the lowest ratio of these inflammatory markers seen with the CD9^+^B cell cultures, even though all the cytokines were increased (**Supplementary Figure 2**).

Although the cytokine production induced by CD9^−^ e-B cells expressing 2.5HIP/I-A^g7^ construct was lower than those of the cultures with CD9^+^ B cells, this was likely to result from the difference in the B-cell phenotypes between CD9^+^ and CD9^−^ e-B cells because the majority of CD9^+^ e-B cells are MZ-like B cells and express higher levels of CD80 and CD86, which also provide co-stimulatory signals for CD4^+^ T-cell activation. Of note, the e-B cells in the co-culture could also release cytokines, which may contribute to the production of the cytokines tested, and it is not possible to differentiate the origin of the secreted cytokines in this experiment.

In summary, these results indicate that both CD9^+^ and CD9^–^ e-B cells can express the 2.5HIP/I-A^g7^ construct, which interact and activate 2.5HIP/I-A^g7^-specific BDC2.5 CD4^+^ T cells. Upon activation with the non-enriched total e-B cells, as well as the CD9^+^-enriched e-B cells, BDC2.5 CD4^+^ T cells express regulatory markers, with CD9^+^ e-B cells tending to induce higher levels of Lag3, Nrp1, as well as anti-inflammatory cytokines IL-10 and IL-4, but also pro-inflammatory cytokines IFN-γ, IL-6, and TNF-α. These results suggest that both CD9^+^ and CD9^–^ e-B cells can interact with antigen-specific CD4^+^ T cells via the 2.5HIP/I-A^g7^ construct, but the timing with which immune-regulatory e-B cell subsets engage with the CD4^+^ T cells could alter the outcomes of CD4^+^ T-cell activity.

### 3.6 Antigen-specific engineered B cells protect NOD mice from adoptively transferred diabetes induced by G9 CD8^+^ and BDC2.5 CD4^+^ T cells

**Figure 7:**
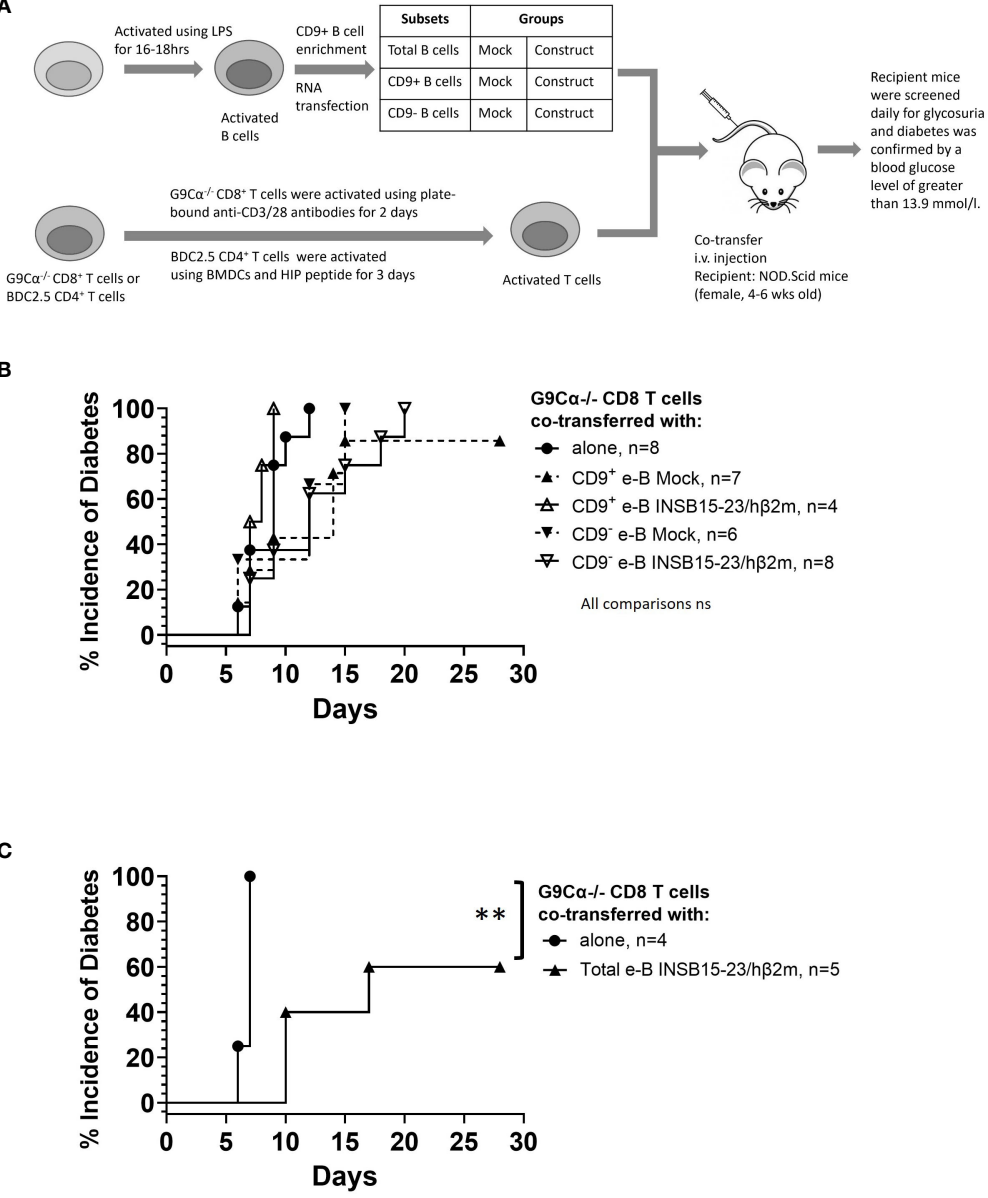
e-B cells delay the development of T1D induced by pathogenic CD8^+^ T cells in NOD.Scid mice. **(A)** Schematic diagram illustrating the *in vivo* adoptive transfer. **(B)** Diabetes incidence in mice co-adoptively transferred at a 1:1 e-B cell:T cell ratio, with the CD9^+^ or CD9^−^ e-B cells either expressing the INS_B15-23_ construct or Mock-transfected (5 × 10^6^ cells) together with insulin-reactive CD8^+^ T cells (5 × 10^6^ cells). Insulin-reactive CD8^+^ T cells (5 × 10^6^ cells) were transferred alone as a control. **(C)** Diabetes incidence in mice co-adoptively transferred at a 5:1 e-B cell:T cell ratio with e-B cells (25 × 10^6^ cells) expressing the INS_B15-23_ construct and insulin-reactive CD8^+^ T cells (5 × 10^6^ cells) per recipient mouse. Insulin-reactive CD8^+^ T cells (5 × 10^6^ cells) were transferred alone as a control. Log rank test **p < 0.01.

Finally, we tested the regulatory function of these engineered B cells *in vivo* for their capacity to protect NOD.Scid mice from diabetes development by adoptively transferring pathogenic antigen-specific CD8^+^ or CD4^+^ T cells into NOD.Scid hosts. The experimental setup is shown schematically in **Figure 7A**.

For the suppression of pathogenic CD8^+^ T cells, insulin-reactive G9 CD8^+^ T cells, isolated from G9Cα^−/−^ mice, were transferred into NOD.Scid mice. Diabetes usually occurs within 12 days after adoptive transfer of the G9 CD8^+^ T cells (24), as was seen in this experiment (**Figure 7B**). When the G9 CD8^+^ T cells were adoptively co-transferred with either antigen-specific CD9^−^ or CD9^+^ e-B cells at a 1:1 ratio, there was a delay in the onset of diabetes, but this delay did not reach significant significance with G9 CD8^+^ T cells plus e-B cells (**Figure 7B**). However, at a 5:1 total e-B-to-T cell ratio, 40% protection was seen (**Figure 7C**) (*p* < 0.01).

**Figure 8:**
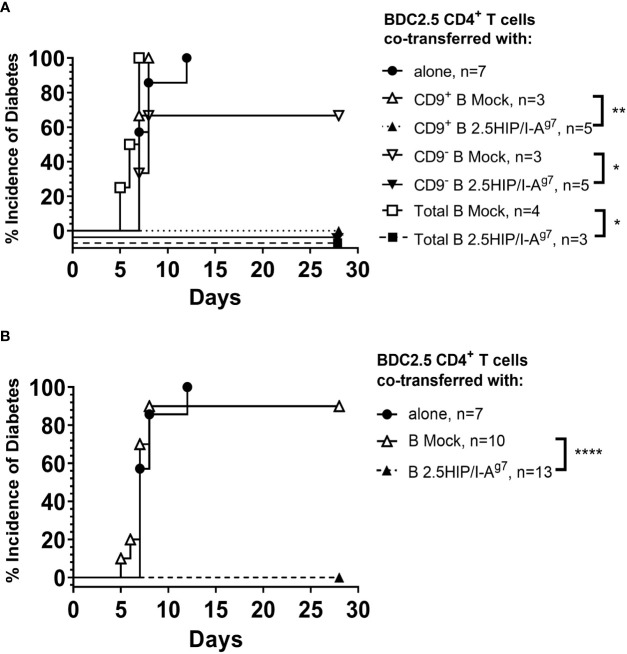
e-B cells prevent diabetes development induced by pathogenic CD4^+^ T cells in NOD.Scid mice. **(A)** Diabetes incidence in mice co-adoptively transferred at a 1:1 e-B cell:T cell ratio, with CD9^+^ or CD9^−^ or total e-B cells either expressing the 2.5HIP/I-A^g7^ or mock-transfected (5 × 10^6^ cells) together with BDC2.5 CD4^+^ T cells (5 × 10^6^ cells). BDC2.5 CD4^+^ T cells (5 × 10^6^ cells) were transferred alone as a control. **(B)** The results from different B-cell groups shown in A were pooled to show overall protection. * p<0.05, ** p<0.01, **** p<0.0001.

For the suppression of pathogenic CD4^+^ T cells, we co-transferred e-B cells that were engineered with the 2.5HIP construct, together with 2.5HIP-reactive BDC2.5 CD4^+^ T cells at a ratio of 1:1. All the recipients of e-B cells, whether CD9^+^, CD9^−^, or unseparated total e-B cells, were protected from the BDC2.5 CD4^+^ T cell-induced diabetes (**Figure 8A** and summarized in **Figure 8B**). No protection was seen on co-adoptive transfer with the control B-cell subsets, which did not express the antigen-specific constructs (**Figure 8A** and summarized in **Figure 8B**).

**Figure 9:**
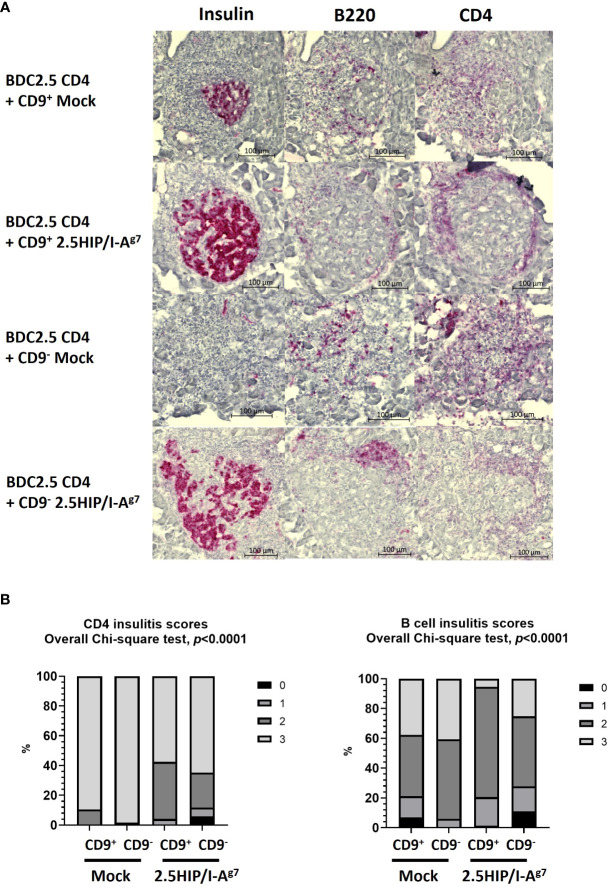
Insulitis scoring of the pancreas tissue from adoptive transfer of mock-transfected or 2.5HIP/I-A^g7^ e-B cells and BDC2.5 CD4^+^ T cells at the end point (diabetes) or 28 days (non-diabetes). All the samples from the mice transferred with mock-transfected e-B cells were diabetic; all the samples from the mice transferred with e-B cells transfected with 2.5HIP/I-A^g7^ were non-diabetic. **(A)** Representative examples of the insulitis within the pancreatic islets from each group, with separate staining (shown as red) for insulin, B cells (B220), and CD4 T cells (CD4). **(B)** Insulitis scores of the mice groups that received BDC2.5 CD4^+^ T cells together with CD9^+/−^ e-B cells with or without the 2.5HIP/I-A^g7^ construct. We examined 73–119 islets from two to three mice in each group: CD9^+^ e-B cell Mock (*n* = 2, 85 islets), CD9^−^ e-B cell Mock (*n* = 2, 116 islets), CD9^+^ B cell 2.5HIP/I-A^g7^ (*n* = 3, 73 islets), and CD9^−^ B cell 2.5HIP/I-A^g7^ (*n* = 3, 119 islets). Insulitis was scored separately for the CD4^+^ T cells and the B cells. Insulitis scores were as follows: 0 = no insulitis, 1 = peri-insulitis, 2 = less than 50% infiltration, 3 = greater than 50% infiltration. Data were analyzed by the Chi-square test.

We further examined the insulitis in the pancreas samples of the co-adoptively transferred BDC2.5 CD4^+^ T-cell e-B cell recipients, when the recipient mice were terminated (either when they developed diabetes or at 28 days post-adoptive transfer). We found that the pathogenic BDC2.5 CD4^+^ T cells trafficked to the islets in both the diabetic and the protected mice (**Figure 9A**), whereas those co-transferred with construct-expressing e-B cells were all non-diabetic (protected), and their islets had a lower severity of infiltration (**Figure 9B**). In contrast, the majority of those control recipients receiving the BDC2.5 CD4^+^ T cells with mock-transfected e-B cells became diabetic, and clearly had more severely infiltrated islets. The e-B cells also trafficked to the islets (**Figure 9A**), but the B-cell insulitis was also significantly less abundant in the protected mice (**Figure 9B**), as shown by the increased lower scores of the insulitis.

## 4 Discussion

In this study, we explored a novel strategy to engineer B cells to express chimeric antigen-MHC constructs that interact with pathogenic islet-autoantigen-specific CD8^+^ and CD4^+^ T cells. We showed that the MHC–antigen–peptide constructs are successfully expressed on the B cells and present the antigenic peptides to their respective antigen-specific T cells (**Figures 1**, **2**). Using a strategy to enrich total B cells for a potentially IL-10-producing regulatory B-cell population, we selected a subset of cells expressing CD9, previously demonstrated to be enriched for IL-10-producing cells (35) (**Figure 3**). *In vitro*, the CD9^+^ B cells suppressed cytotoxicity of insulin-reactive CD8^+^ T cells, although this was not enhanced by the expression of the antigen-specific construct (**Figure 4**). *In vivo*, the e-B cells expressing the antigen-specific construct recognized by pathogenic CD8^+^ T cells were able to protect against diabetes adoptively transferred by insulin-specific CD8^+^ T cells into immunodeficient NOD.Scid mice at a high B cell-to-T cell ratio (5:1). Although the protection was not obvious at a much lower ratio of B-to-T cells (1:1), there was a potentially promising possibility (**Figure 7**). The 2.5HIP/I-A^g7^construct expressing e-B cells, regardless of CD9 expression, induced the expression of immune regulatory markers on diabetogenic CD4^+^ T cells (**Figure 5**), and in marked contrast to the CD8^+^ T-cell transfers, when the engineered B cells were co-transferred with BDC2.5 islet-reactive pathogenic CD4^+^ T cells into NOD.Scid mice, these e-B cells notably protected all the recipients (**Figure 8**), and significantly decreased the severity of insulitis (**Figure 9**).

We have previously used a modified CAR strategy to endow T cells with the ability to target both CD4^+^ and CD8^+^ T cells (20, 21, 30). Here, we took a similar approach to generate e-B cells that can be recognized by antigen-specific CD4^+^ and CD8^+^ T cells. The immunoglobulins expressed by B cells, as the only antigen-specific antigen-presenting cells (compared with macrophages and dendritic cells), naturally present their bound antigens specifically to T cells. However, identifying and purifying large numbers of naturally occurring antigen-specific B cells is not an easy task, and is not practically possible. Our approach increased the antigen-specific potential of B cells and, at the same time, sought to utilize the functional regulatory properties that are present in some subsets of B cells (37, 38). LPS is often used to stimulate B cells, and it is known that LPS-stimulated B cells produce the anti-inflammatory immune regulatory IL-10, and LPS-stimulated B cells can protect NOD mice from autoimmune diabetes (9). To further enhance the regulatory potential of the CAR e-B cells, we sought to further increase IL-10-expressing B cells, using CD9 to enrich for IL-10-producing B cells (35). With this strategy, we found that 40% of CD9-enriched B cells expressed IL-10 in the population, and we suggest that CD9 could be used as a Breg marker to enrich for IL-10^+^ B cells. This CD9 enrichment is much simpler and does not require the amount of manipulation of the cells that is used in other Breg purification methods, which include re-stimulation of the cells after selection. Thus, we engineered IL-10-enriched B cells to express antigen-specific constructs.

The first set of e-B cells, expressing INS_B15-23_/hβ_2_m, were recognized together with H-2-K^d^ by pathogenic INS_B15-23_-specific G9 CD8^+^ T cells. The second set of e-B cells, expressing 2.5HIP/I-A^g7^, were recognized by pathogenic 2.5HIP/I-A^g7^-specific BDC2.5 CD4^+^ T cells. These e-B cells allowed us to specifically test, as a proof of principle, the ability of antigen-specific IL-10-producing e-B cells to interact with CD8^+^ and CD4^+^ antigen-specific T cells and protect from aggressive antigen-specific induced autoimmune diabetes.

In adoptive transfer, the insulin-specific CD8^+^ T cells are highly pathogenic; they require pre-activation in order to cause diabetes, and normally diabetes is adoptively transferred in 7–14 days. Here, we showed that at a 1:1 INS_B15-23_/hβ_2_m e-B cell-to-CD8^+^ T cell ratio, irrespective of whether the e-B cells were CD9^+^ or CD9^−^ or whether the e-B cells expressed the antigen-specific construct, they were ineffective in delaying or preventing adoptively transferred diabetes by fully activated antigen-specific CD8^+^ T cells (**Figure 7B**). Interestingly, at the higher ratio of total e-B cells to CD8^+^ T cells (5:1) (**Figure 7C**), there was significant protection in the small number of recipients tested. Although this was a potentially promising avenue for exploration, the number of cells required for either further experiments with the 5:1 total e-B:T cell ratio or co-transferring the CD9^+^ or CD9^–^ e-B cell subsets at the 5:1 B-to-T cell ratio was prohibitively large to be practical, and we were not able to further test for this.

However, in the future, it would be interesting to test whether e-B cells introduced into young prediabetic mice in the early stages of development of diabetes, before the full activation of antigen-specific CD8^+^ T cells, may be more efficient in protection. We could also potentially increase interaction with CD8^+^ T cells *in vivo* by introducing other genes that could include chemokine receptors. Our *in vitro* testing indicated that CD9^+^ INS_B15-23_/hβ_2_m e-B cells had some effect in reducing the development of cytotoxicity of G9 insulin-reactive CD8^+^ T cells towards their targets in cytotoxicity assays (**Figure 5B**) (rather than inhibiting the fully activated CD8^+^ T cells shown in the *in vivo* transfer). However, this was also seen with the non-transfected CD9^+^ e-B cells, and thus, the addition of the antigen-specific construct did not further add to cytotoxicity reduction. However, in the *in vitro* assay, all the cells are in very close proximity, and any cell–cell interactions could occur whether or not the cells were drawn together with the specific construct recognition.

BDC2.5 CD4^+^ T cells, recognizing the 2.5HIP antigen, are also highly diabetogenic, transferring diabetes to NOD.Scid mice in 7–14 days. In contrast to the results seen with the CD8^+^ T cells, co-transfer of the e-B cells expressing the 2.5HIP/I-A^g7^ construct, together with pre-activated pathogenic BDC2.5 CD4^+^ T cells, completely protected the NOD.Scid recipient mice from developing diabetes, regardless of which subset of B cells was used (**Figure 8**). The histology of pancreatic islets in the mice protected from diabetes development (those co-transferred with each of the e-B cell subsets expressing 2.5HIP/I-A^g7^construct) showed that CD4^+^ T-cell and B-cell infiltration still occurred, but as peri-insulitis, and the islet-β cells still maintained the production of insulin (**Figure 9**). These data suggested that despite the infiltration, the islets were less damaged (**Figure 9**), and we may speculate that some of the protection may occur actually in the islets. As this effect occurred for both the CD9^+^ and CD9^−^ e-B cell groups, as well as total e-B cell group, it suggested that the protection was not CD9^+^ B cell dependent, and that even the total B-cell populations had sufficient regulatory capacity. However, protection was highly dependent on the antigen-specific interaction. As all the B-cell subsets had pre-activation with LPS in preparation for 2.5HIP/I-A^g7^construct engineering, it is likely that other B regulatory cell subsets might also have been activated/expanded, and this, together with the antigen-specificity, was sufficient to protect the islets from the damage by the pathogenic CD4^+^ T cells. Furthermore, it is possible that other regulatory subsets may protect using different suppressive mechanisms, a possibility to be considered in future experiments.

In considering the mechanism by which the antigen-specific e-B cells can regulate naïve CD4^+^ T-cell responses, we found that the e-B cells increased cell surface proteins (PD-1, LAP, Nrp1, ICOS, and CTLA-4) associated with regulatory function on CD4^+^ T cells following co-culture *in vitro* (**Figure 3**). It is possible that following interaction with the B cells, the infiltrating CD4^+^ T cells have become less inflammatory. The time course of the expression of these regulatory proteins, particularly Lag3 and Nrp1, in CD4^+^ T cells cultured with the CD9^+^ e-B cells transfected with the 2.5HIP/I-A^g7^ construct, appeared to be different. This observation could be important in future experiments utilizing these antigen-specific e-B cells administered to younger pre-diabetic NOD mice to test their ability to prevent spontaneous diabetes at the optimal time. We also observed that the cytokine production by BDC2.5 CD4^+^ T cells upon activation differed between cells co-cultured with CD9^+^ e-B cells and CD9^−^ e-B cells (**Figure 6**). In keeping with other observations showing that in order to exert regulatory activity, regulatory T cells may co-express inflammatory cytokines (39–41), it was interesting to also observe this phenomenon here, when the BDC2.5 CD4^+^ T cells were stimulated by the e-B cells. These results provide an alternative antigen-specific strategy to other means of protection against pathogenic CD4 cells as has been recently shown using nanoparticles containing 2.5HIP (42).

In considering why the strategy of using e-B cells to inhibit pathogenic BDC2.5 CD4^+^ T cells was more successful than the inhibition of pathogenic G9 insulin-reactive CD8^+^ T cells, there are a number of possible explanations. We and others have shown that while the CD8^+^ T cells can interact directly with B cells (43), we have also demonstrated that Breg inhibit G9 CD8^+^ T cells by a mechanism of inducing tolerogenic dendritic cells, which, in turn, inhibit the G9 CD8^+^ T cells (44). Thus, it is likely that there are differences in the ability of B cells to directly induce tolerance in CD8^+^ T cells compared with the CD4^+^ T cells. In addition, the constructs themselves are different regarding the presentation to the T cells. The INS_B15-23_/hβ_2_m construct utilizes the expression of native MHC I alpha chain on B cells, as it consists of hβ_2_m with the linker, which means that the level of construct molecule may be lower than the 2.5HIP construct, which consists of both MHC II chains together with the peptide. We cannot directly measure the expression of the 2.5HIP construct, but based on the activation of the CD4^+^ T cells (**Figure 2B**), compared with the activation of the CD8^+^ T cells (**Figure 1D**), there is greater activation of the CD4^+^ T cells, and a greater potential for increased induction of tolerance.

We have obtained *in vitro* and *in vivo* proof of concept for our approach by expressing the chimeric MHC peptide construct in NOD B cells using mRNA electroporation. Transfection of mRNA to modify primary human and mouse T cells has advantages in that electroporation of mRNA is fast, simple, and efficient and drives high and uniform expression under mild conditions, thereby preserving cell viability. Although transient, mRNA transfection can drive functional expression of the introduced genes for sufficiently long to have a biological effect. The short interaction will bring the B cells and T cells into close proximity, and this may also allow continuation of any regulation or alteration to T-cell phenotype, related to the regulatory B-cell phenotype that is not dependent on construct expression. We assume that under such circumstances, the actual number of e-B cells required for suppressing the diabetogenic T cells far exceeds the required number of e-B cells that would express the chimeric products stably (using viral or non-viral methods). Our next steps would be to move to the non-immunodeficient wild-type NOD model where we may require more than one transfer of e-B cells. Any such strategy is likely to require more than one administration of cells. We could also test the use of more stably transformed B cells using viral vectors in a future study.

Taken together, we reveal the potential of B cells that have been stimulated to increase regulatory cytokines to be engineered and manipulated using our chimeric autoantigenic peptide/MHC constructs, in order to strengthen the engagement of islet antigen-specific CD4^+^ and CD8^+^ T cells. The regulatory e-B cells can express and present the constructs to the islet antigen-specific T cells. Importantly, our *in vivo* co-transfer experiments have demonstrated the protective role of these specific antigen–peptide construct-expressing regulatory e-B cells, indicating the potential for translation of this approach as a novel immunotherapy, not only for T1D, but also possibly for other autoimmune diseases. To our knowledge, this is the first time the expression of this type of modified chimeric antigen receptor (CAR) in B cells with regulatory properties has been shown to be effective in autoimmunity. There are still questions to be answered and future investigation would include assessing whether these antigen-specific e-B cells can protect NOD mice from development of spontaneous diabetes, when antigen-specific e-B cells are transferred at both early and late phases of the natural history of autoimmune diabetes. It is possible that e-B cells expressing antigens or peptides recognized by both CD4^+^ and CD8^+^ T cells may be required. With this, we would also further investigate other methods of enrichment for regulatory B-cell types, and our strategy suggests that generating antigen-specific e-B cells is a novel way to further enhance regulatory B cells, to protect against T1D.

## Data availability statement

The original contributions presented in the study are included in the article/**Supplementary Material**. Further inquiries can be directed to the corresponding author.

## Ethics statement

The animal study was approved by UK Home Office/Cardiff University Biological Standards Committee. The study was conducted in accordance with the local legislation and institutional requirements.

## Author contributions

The study was conceived and designed by GG and FSW. DC, DK, SF, HW-M, JD, JB, and TT performed experiments. GG and FSW supervised the study. DC and FSW wrote the manuscript. DC, DK, JB, LW, GG, and FSW edited the manuscript and all authors approved the final manuscript.
