# Supplementary material for: Novel engineered B lymphocytes targeting islet-specific T cells inhibit the development of type 1 diabetes in non-obese diabetic Scid mice
Source: Front Immunol. 2023 Sep 4;14:1227133. doi: 10.3389/fimmu.2023.1227133 (PMC10507356; doi:10.3389/fimmu.2023.1227133)
Supplement: Supplementary file 1 [file DataSheet_1.docx]

**Supplementary figure legends**

**Supplementary Figure 1**. Proliferation of BDC2.5 CD4^+^ T cells co-cultured with different types of e-B cells transfected without (Mock) or with 2.5HIP/I-A^g7^ construct over 3 days. BDC2.5 CD4^+^ T cells were labelled with CellTrace Violet and co-cultured with e-B cells at B cell: T cell 1:1 ratio. A. CD4+ T cell proliferation was determined by dilution of CellTrace violet. Proliferated CD4^+^ T cells were gated and the frequencies of proliferated cells are plotted in the line-graph (B), shown as the mean±SEM of 5 independent experiments. Statistical analysis was done by 2-way ANOVA with Multiple Comparisons. +*p*<0.05, ++*p*<0.01, ++++*p*<0.0001, comparisons between 2.5HIP/I-A^g7^ construct vs. mock transfected within each B cell group are shown in colour on the graph.

**Supplementary Figure 2**. Ratios of levels of individual cytokines vs. the level of IL-10 production after co-culture of e-B cells and antigen-specific BDC2.5 CD4^+^ T cells. Levels of each cytokine were measured as described in Figure 6. The ratios of cytokines against IL-10 were calculated using the levels of A. IFN-γ B. IL-4 C. IL-6 D. TNF-α, divided by the level of IL-10 from the same samples. Statistical analysis was performed using 2-way ANOVA with Multiple Comparisons. Significance was determined by the *p* values using Multiple Comparisons between B cell types with 2.5HIP/I-A^g7^ construct. ns non-significant, * *p*<0.05, ***p*<0.01, ****p*<0.001, *****p*<0.0001.
