# Supplementary figures and images for: Novel engineered B lymphocytes targeting islet-specific T cells inhibit the development of type 1 diabetes in non-obese diabetic Scid mice
Source: Front Immunol. 2023 Sep 4;14:1227133. doi: 10.3389/fimmu.2023.1227133 (PMC10507356; doi:10.3389/fimmu.2023.1227133)

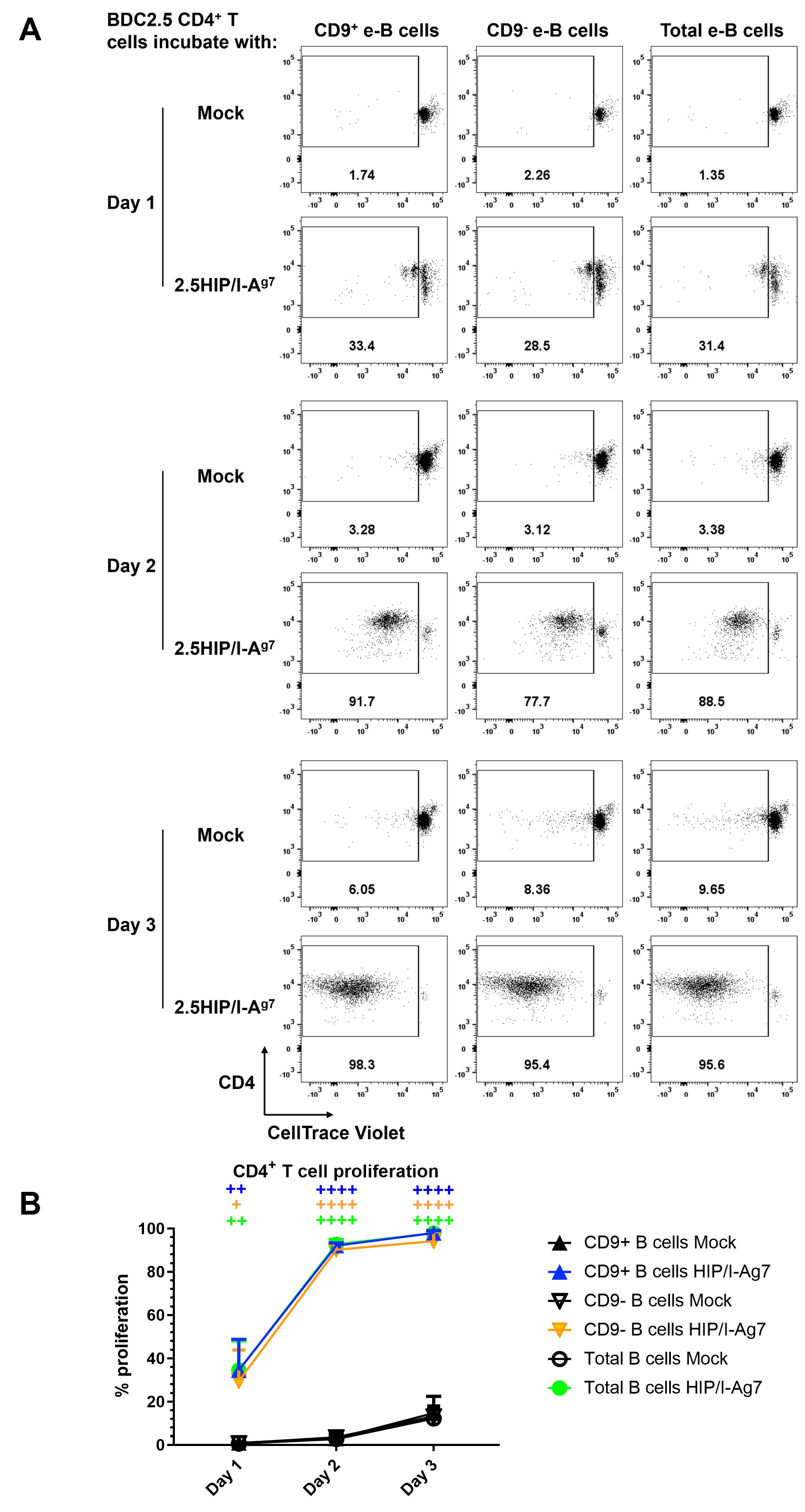

Supplement: Supplementary file 2 [file Image_1.jpg]

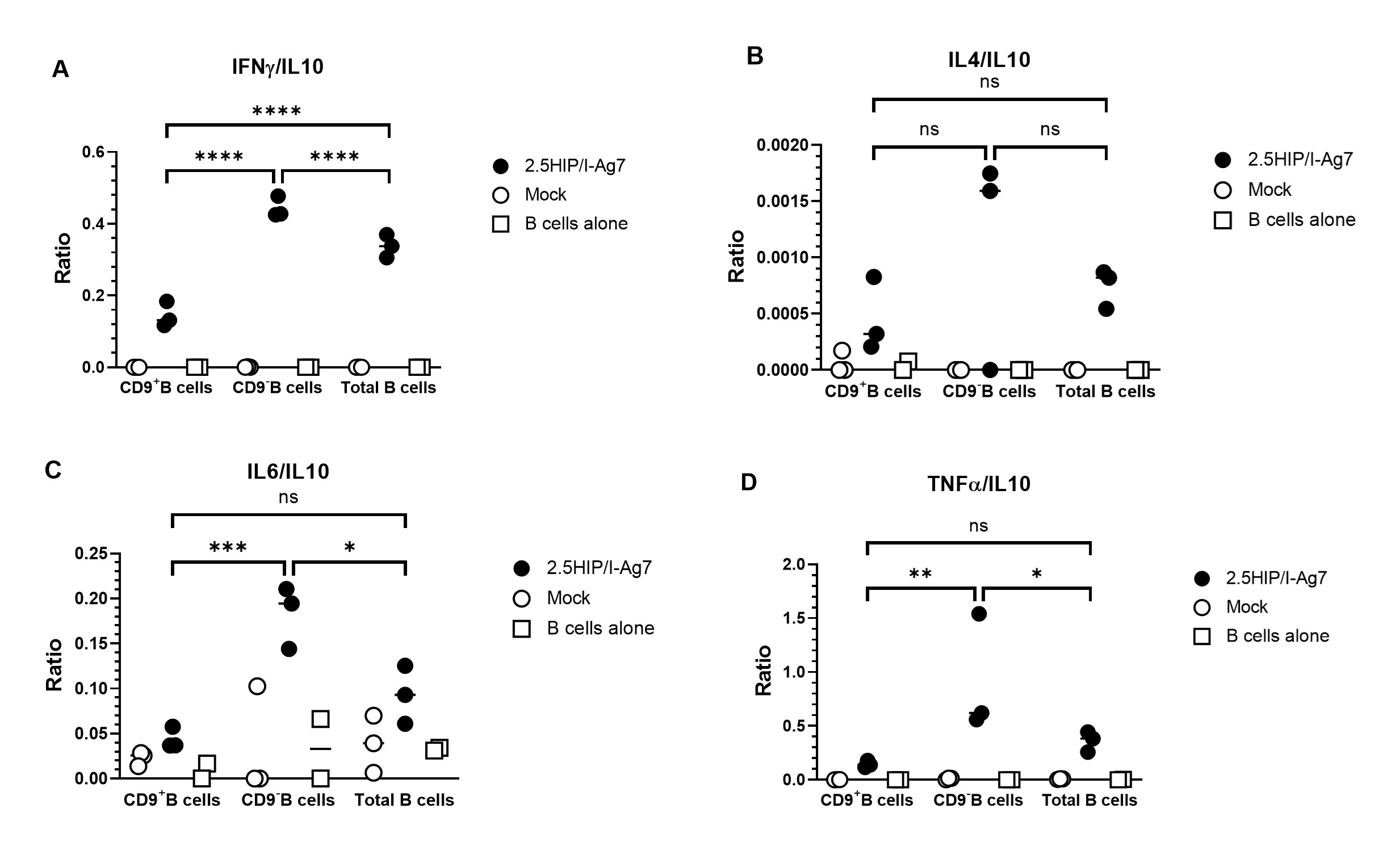

Supplement: Supplementary file 3 [file Image_2.jpg]
